# Motivations and Specialization of Birders Are Differently Related to Engagement in Citizen Science Projects of Different Complexity

**DOI:** 10.3390/bs12100395

**Published:** 2022-10-16

**Authors:** Christoph Randler

**Affiliations:** Department of Biology, Eberhard Karls University Tübingen, Auf der Morgenstelle 24, D-72076 Tübingen, Germany; christoph.randler@uni-tuebingen.de

**Keywords:** citizen science, recreation specialization, ornithology, birding, birdwatching, monitoring

## Abstract

Citizen Science (CS) projects are an important aspect of scientific data collection and biodiversity conservation. In ornithology, various CS projects exist, and even laypersons can contribute, but advanced birdwatchers also spend considerable time and effort in data collection. Here, different CS projects for birders were analyzed and compared with respect to recreation specialization and motivations for birdwatching. Established, psychometrically valid, and reliable scales were applied in this study. N = 2856 respondents from Austria, Germany, and Switzerland were grouped into no, low, and sustained engagement clusters. Sustained engagement was related to more complex programs, such as the breeding bird census and waterfowl counting. When comparing the engagement clusters, effect sizes were considerable, ranging from 0.098 (attraction) to 0.306 (skill/knowledge). Thus, birders of the three engagement clusters differed significantly in birding specialization, especially skill/knowledge, psychological commitment, social motivations, and the psychological construct centrality to lifestyle. No differences were found in enjoyment and achievement motivations. In conclusion, low-threshold projects are needed to attract participants, but keeping people within programs or moving them to a higher level of engagement might be easier when social dimensions are addressed.

## 1. Introduction

Citizen Science (CS) projects have become increasingly important aspects of scientific data collection because they allow for the study of large-scale phenomena in nature [1,2]. In addition, these data can be further used for biodiversity conservation and monitoring [3]. Some projects would not be possible without the engagement of citizen scientists because they would cost millions of dollars if data could be collected only by educated and trained scientists [1].

One example of successful CS is in ornithology. Here, even laypersons can contribute easily, but advanced birdwatchers also spend considerable time and effort in data collection, even of rare and endangered species [1,4,5]. CS projects in ornithology are available on different knowledge, specialization, and involvement levels. More basic and low-involvement projects include, e.g., feeder watch programs or the Christmas Bird Count that can be based on a single time point in data collection [6,7]. Haphazard and more convenient programs are web-based platforms where birders can contribute ad libitum, such as eBird, but usually many people provide large datasets over longer periods of time [4,8]. Further projects include long-term waterfowl monitoring or ringing projects, where commitment over longer periods of time, sometimes over decades, is encouraged. Some projects even request a special education/training where participants obtain a license before they can start contributing, such as constant effort ringing programs where birds are caught with mist nets and ringed [9].

Birding is a widespread nature-related outdoor leisure activity, and many birdwatchers spend their valuable spare time on data collection for conservation [4,10]. However, to make the data even more valuable for conservation, it is important to learn more about the citizen scientists themselves [8,10,11]. Similarly, knowledge about birding specialization and motivations can help to address and target birdwatchers and to foster their contribution to specific citizen science programs.

Although CS projects are thriving, there is still a dearth of knowledge about the citizen scientists themselves. Some exceptions exist. Sustained participation in combination with regular reporting of observations increases the identification skills of the participants [8,12]. A sample of citizen scientists was more engaged in sharing information and in teaching youth than non-citizen scientists [13]. The citizen scientists also reported improved science process skills and a better understanding of the nature of science [13]. Knowledge and competencies were reported as a benefit for participation [14,15,16], and interest in and understanding of science were improved [17].

Concerning motivation, there are some previous studies on motivational aspects in green volunteering CS projects. Hobbs and White [18] highlighted the major motivating factors for the participation in wildlife recording schemes as the chance to make a positive contribution to conservation and personal benefits. Guiney and Oberhauser [19] emphasized the importance of the connection to nature, which often started during childhood for the citizen scientists. Ganzevoort and van den Born [20] and Ganzevoort et al. [21] showed that having a connection to and interest in nature were important aspects, as were concern for nature and desire to contribute to conservation. In birding, participants of the Christmas Bird Count reported different motivational dimensions: science/conservation, outdoor recreation, commitment, social interaction, classic birding, and personal accomplishment [7]. Thus, motivation to participate in different CS programs may differ between the participants’ groups because birders are a heterogenous group [10]. To better understand birders as citizen scientists, apart from motivation, the recreation specialization concept is necessary.

Recreation specialization is a conceptualization that was developed by Bryan [22] in the case of trout fishermen. He defined the specialization as a leisure career. In line with Bryan’s [22] conceptualization, birdwatchers can be classified along a continuum of specializations from the novice to the advanced birder [10,23]. Novices have a lower involvement in terms of investment of time and money and usually have lower skills, while highly specialized birders show a high level of commitment [24]. Although birdwatchers can be classified along a continuum based on a metric score [25], some approaches have grouped them into four distinct groups: casual birder, novice, intermediate, and specialist/advanced birder [23]. The measurement of recreation specialization is usually based on a multidimensional assessment with three dimensions: skill, commitment, and behavior [26]. Skill measures the ability to identify bird species by sight and call/songs. Behavior measures the number of birding trips, the number of species on a life list or national list, and money invested in equipment, trips, and books [10,23,26]. Commitment is understood as a psychological variable, measured as personal and behavioral commitment. This includes engagement in a leisure activity. Furthermore, commitment is related to centrality to lifestyle [27]. In general, commitment and centrality to lifestyle measure the importance of the birdwatching hobby within a person’s lifestyle. The recreation specialization concept has not yet been studied in relation to CS programs.

In addition to specialization, birding motivations are a further psychological approach to understand birdwatchers [23]. Apart from identifying and seeing birds, motivations can also be related to a diverse array of social, psychological, emotional, and physical benefits [28]. Decker et al. [29] postulated that there are three broad dimensions of motivations in relation to outdoor recreation: first, an affiliative component which is related to the social dimension (e.g., meeting people with the same interest); second, an achievement orientation (or competition) where performance is important (number of bird species recorded); and third, an appreciation or enjoyment-oriented motivation such as enjoying the peacefulness of nature or being impressed by the aesthetics of birds. This model is well-established in outdoor recreation and was adapted from general outdoor recreation to birders [11,23]. Some studies found and defined an additional, separate dimension, labelled conservation [7,23,24,30]. The Decker et al. [29] model for motivations in outdoor recreation was chosen for this study because it has been applied to birding since 1994 [23] and it has a solid factor structure, i.e., the model is supported by a psychometrically well-established scale. Thus, as this scale was successfully developed for birders, follows a solid theoretical underpinning, and has a sound psychometric basis, and birders are the target group of this research on CS, it was deemed an optimal choice.

Many studies have been carried out during the last few decades to characterize the motivations and specializations of birders [23,24,25,30,31,32]; further, motivational studies were linked to CS participation [18,19,20,21,33], while, in turn, the analyses of citizen scientists in ornithology were not linked with the recreation specialization concept. As there are already valid and reliable scales available for birding activities, this study draws on the analyses of recreation specialization and motivations in relation to CS birding programs because neither the specialization nor the motivations have been related to the variety of CS programs available for birdwatchers. The aim of this study was to assess specialization levels and motivations in birdwatchers and relate them to their differential engagement in CS projects.

## 2. Material and Methods

### 2.1. The Different CS Programs

CS projects reach from data collection to data processing, to curriculum projects, to community science [17]. Different CS programs were studied here, but they can be grouped largely within the data collection dimension of CS. The largest nature conservancy (NGO) in Germany is Naturschutzbund Deutschland (NABU, with the associated Bavarian chapter LBV). Two very common programs are available for birders. During the wintertime in January, there is a weekend for garden birds/feeder birds; during a weekend in May, there is a data collection period for summer garden birds. Both programs request the participants to watch one patch, usually one’s own garden or surroundings for one hour and note the highest count of every bird species. The programs are supported by the media (online, print) and by the local chapters of the NGO. These activities represent a low threshold for participants and are available for beginners. The time effort needed is about 1 h (and additional time for reporting the results by mail or web-based tools). During 2021, there were 236,554 participants for the winter bird count and 140,000 in the garden bird count (May). Another low threshold activity is the cleaning and maintenance of nest boxes, which is usually also organized by the nature conservation associations. During this activity taking place during and after the breeding season, data are collected about nest box occupancy (species determination) or breeding success/failure, and these data are used for monitoring studies. However, this requires a little more time, and often is a regular reoccurring activity done every year. There are no data about how many people participate in this activity. More complex programs are related to monitoring programs of the DDA (Dachverband Deutscher Avifaunisten). These programs are usually designed for regular yearly participation in either the waterfowl census (between 1 and 8 times per winter season; September to April; carried out at 6000 different places) or the breeding bird surveys (4 times per spring),. The breeding bird census is carried out on 2637 patches. They request more time in the field, as well as higher knowledge and also some paperwork. The breeding bird census is more demanding because waterbirds comprise fewer species and can be easily identified in winter, while breeding bird counts very often rely on acoustic cues. Most sophisticated is the contribution to bird ringing schemes. To carry out bird ringing, the scientist needs a special course and education to receive a license. Furthermore, bird ringing demands some specific knowledge and requires a lot of time in the field. The constant effort of ringing requires about 12 sessions per year with some hours for netting and ringing birds.

### 2.2. Data Collection

Data were collected by an online research tool (SoSciSurvey) in Austria, Germany, and Switzerland to cover the German-speaking adjacent countries (only the German speaking parts of Switzerland were surveyed) because they are assumed to have similar birding behaviors, and cross-border birding, e.g., in the Lake Constance region, is very common. Moreover, they can use the same digital tools and web-based information because there is no language barrier. Data collection took place between 14 February 2020 and 15 June 2020. In the beginning, the goals of the study were explained, and formal informed consent was requested. Participants had to actively click “yes” to start the study. The aim of the recruitment procedure was to cover a wide range of birders from different organizations, ranging from people preferring backyard birdwatching to highly specialized birdwatchers (club300). Many channels were used, including using announcements on webpages of large bird and nature-related organizations and from regional chapters of scientific ornithological unions, societies, and clubs. In addition, an advertisement was placed in a printed birdwatching journal. The study was granted permission from the ethics committee of the Social Science and Economics Faculty of the University of Tuebingen (Az A2.5.4.-113_aa). The data that support the findings of this study are available on request from the corresponding author.

#### 2.2.1. Assessment of CS Participation

Participants were asked for their participation in different CS projects related to birds. All projects were presented with a response format ranging from 1 = never, 2 = one time, 3 = 2–3 times, 4 = 5–10 times, and 5 = >10 times. The categories were winter birds, garden birds, waterbird counts, breeding birds, ringing, and nest boxes.

#### 2.2.2. Birding Specialization

Birding specialization was measured with the three-dimensional construct provided by Lee and Scott [26]: skill, behavior, and commitment. Skill was measured by the number of species a person was able to identify without a field guide by appearance and by song, supported by a self-assessment of knowledge from 1 (novice) to 5 (expert) [26]. Behavior was measured by the number of birding trips taken in the last year (at least 2 km away from home); number of days spent for birding last year, number of bird species on their life list, number of bird books owned, replacement value of the total equipment, and number of species on a national list [10]. Personal commitment was measured with items such as: “Other leisure activities don’t interest me as much as birding.” Behavioral commitment was based on three items (e.g., “If I couldn’t go birding, I am not sure what I would do.”). Involvement was assessed with the modified involvement scale [27]. Three items each measured “attraction”, “centrality to lifestyle”, and “social bonding”. Finally, identity was measured with one item (“Birdwatching is an important part of my identity.”) [34]. For the details of the scales, see [10].

#### 2.2.3. Motivations for Birdwatching

Fourteen motivation items were used to measure motivations, covering the three dimensions of motivations: social, competition/achievement, and enjoyment of nature [11]. These three dimensions are based on the model proposed by Decker et al. [29] and adapted by McFarlane [23] for birdwatchers. The items are summarized to build a psychometrically valid scale [11]. The items measuring motivations were coded from 1 = fully disagree to 5 = fully agree. Higher scores refer to higher motivation. An example for the social dimension is “…meet people who share my interests” (N = 6 items); an example for the competition/achievement dimension is “…seeing as many bird species as possible.” (N = 2 items); and finally, an example for the enjoyment dimension is “enjoy being alone” (N = 6).

#### 2.2.4. Demographics

Respondents were asked about their demographics (age in years; gender: male, female, diverse, prefer not to answer).

### 2.3. Statistical Analysis

To analyze the heterogeneity of the different participants concerning their contributions to the various CS projects, a two-step cluster analysis was applied. Data are often heterogenous and can be grouped by cluster analysis into homogenous groups. Cluster analysis uses multivariate techniques to minimize the distances between cases within a group (intra-group homogeneity) against the higher distances between groups (inter-group heterogeneity; [35]). Two-step cluster analysis is especially designed to process many data and can apply different measurement levels [35]. The two-step cluster analysis suggests cluster solutions based on the data, saves the suggested cluster for each case as a new variable, and the silhouette variable is used as a kind of measure for cohesion and separation. To assess the differences between clusters in relation to specialization and motivation, first, a multivariate linear model was used, and only the significant results (see below) were considered in the subsequent univariate analyses. For demographical comparisons, a multivariate linear model was applied. Only significant differences on the 0.01 level were considered. In addition, only partial et-squared as a measure of effect size > 0.05 were considered meaningful. SPSS 27 was used for all analyses.

## 3. Results

From all respondents, N = 2856 gave answers concerning their activities in a specific CS program, of which 1508 were male, 1334 were female, and 14 were diverse. The mean age was 47 ± 16 years (SD). Table 1 shows the relationship of the different CS programs with each other. The most common programs were the garden and winter bird counts from NABU and LBV, along with the nest box schemes.

**Figure 1:**
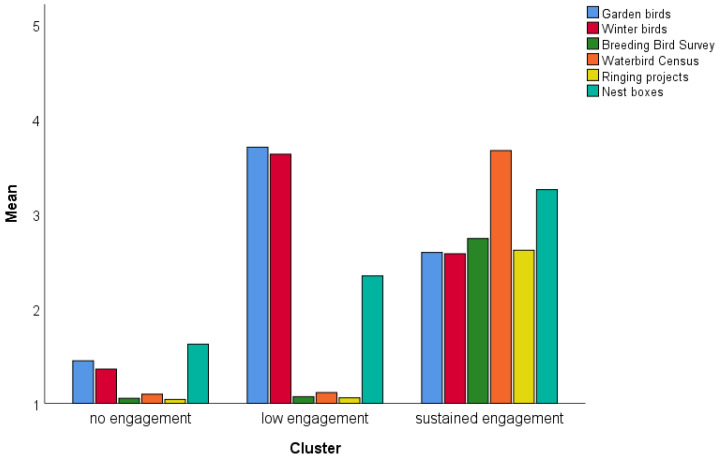
Results of the two-step cluster analysis. The y-axis shows the mean of the cluster for every variable. A high value represents a high engagement in the respective CS program.

The scores of the six different CS programs were used as variables to predict the clusters. The two-step cluster analysis suggested a three-cluster solution. The silhouette variable was acceptable with 0.4, suggesting an acceptable fit of the three-cluster solution [36]. The cases/individuals were automatically grouped into one of the three clusters. The three engagement clusters are depicted in Figure 1. An ANOVA showed significant differences between all three clusters concerning the six CS programs (*p* < 0.001 in all cases; see Figure 1).

**Figure 2:**
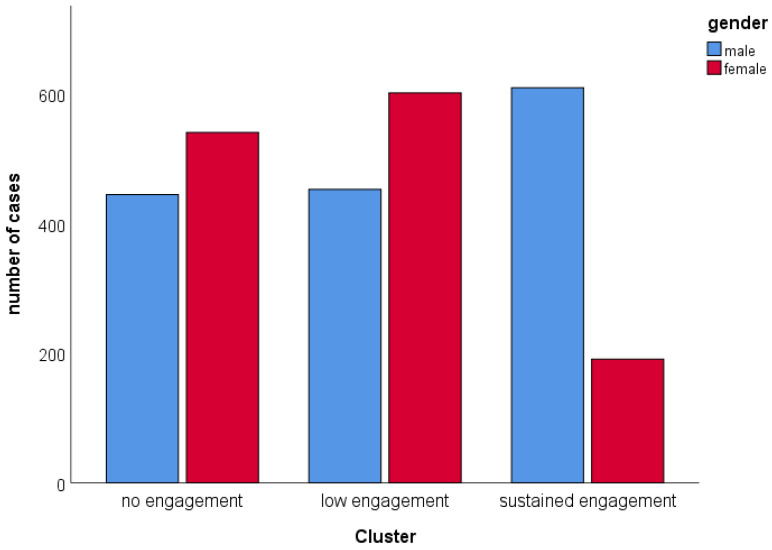
Gender comparison concerning the three clusters of engagement in CS projects.

The engagement clusters can be labeled according to the engagement in the CS program, with cluster 1 as no engagement (N = 992), cluster 2 as low engagement (N = 1058), and cluster 3 as high or sustained engagement (N = 806). Participants from the low engagement group participated in the garden and winter birds counts, but at a significantly higher level than the sustained engagement cluster. The sustained engagement cluster is classified by a sustained engagement in the more complex and enduring programs like the breeding bird survey, bird ringing schemes, and waterbird census. However, people from the higher engagement cluster showed lower investment in garden bird and winter bird counts compared to the low engagement cluster. There was a significant gender effect (chi-square test, χ = 239.814, df = 2, *p* < 0.001, Figure 2), with more men than women showing a sustained engagement.

To further analyze the differences in motivations and specialization levels of birders concerning the different engagement clusters, a multivariate linear model was applied, and the significances and partial eta-squares were checked to inform further subsequent univariate models. Wilk’s λ was 0.614, F = 66.84, *p* < 0.001, and partial eta-squared was 0.22. Thus, the full model was significant and had a high effect size.

The differences between engagement clusters were significant in all dimensions, except in competition motivation (Table 2). In addition, only explained variances with a partial eta-squared > 0.05 were considered. These results show negligible differences in competition and enjoyment motivations, suggesting that the attrition to CS programs is independent of the motivations to be outdoors and enjoy birds (enjoyment motivation), and is also not influenced by the competitive component, e.g., seeing as many bird species as possible. Social motivation was significantly different between the engagement clusters, with the highest scores in the sustained engagement cluster (Table 3).

**Figure 3:**
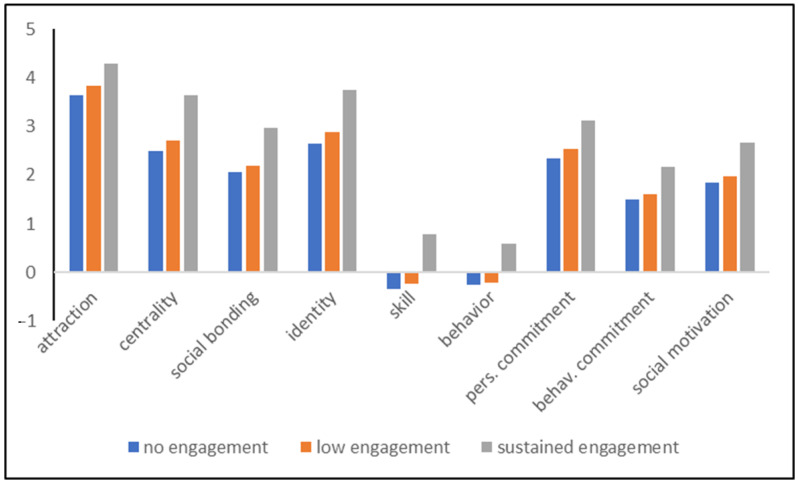
Mean values of the different scales according to the three engagement clusters of CS programs.

Partial eta-squares were quite high in the other dimensions, ranging from 0.098 in attraction to 0.263 in behavior and 0.306 in skill/knowledge. Post-hoc tests on the means were carried out with Bonferroni adjustments (Table 3, Figure 3). All pairwise comparisons between the high engagement cluster and the no engagement cluster were significant on the 0.001 level, as were the differences between high and low engagement clusters. There were no differences in behavior between the low and no engagement clusters (*p* = 0.432).

The demographic analysis of motivations was based on a general linear multivariate model with age as the covariate, gender and country as independent factors (predictors), and the three motivation scales as dependent variables. The model revealed no significant interaction between country and gender (*p* > 0.45). Age showed a significant effect (Wilk’s λ was 0.934, F = 66.42, *p* < 0.001, partial eta-squared = 0.066). Motivation decreased with increasing age. Correlations between age and motivation were −0.107 for social motivation, −0.232 for achievement, and −0.179 for enjoyment (all *p* < 0.001). Concerning gender, there was a significant difference (Wilk’s λ =0.985, F = 13.95); however, the partial eta-squared was 0.015, which is a negligible effect size. Similarly, country produced a significant effect with a negligible effect size (Wilk’s λ =0.991, F = 4.40, partial eta squared = 0.005).

## 4. Discussion

Most participants in our survey participated in the low-demand activities (garden birds, winter birds; Table 1), while the more complex programs like ringing, breeding bird survey, and waterbird count were less common. This shows that easier projects with a low involvement and lower sustained engagement can attract many birders. However, short online programs usually have low retention of participants [16].

The strongest differences existed in specialization, especially in skill/knowledge. This was somewhat predictable because sustained engagement requires high skill in bird identification, e.g., in the breeding bird census. Many bird species are primarily located and counted by their vocalizations. In addition, more committed birders are more engaged [37]. Interestingly, there was no difference concerning behavior, i.e., the number of field trips and excursions, between the three engagement clusters. This may be owed to the fact that the number of field trips is more restricted by work and/or study schedules, which cannot be influenced by the participants. Nevertheless, this study shows that skill is an important predictor for participation in CS programs and should not be neglected in further studies.

Concerning the centrality to lifestyle, all aspects show that a higher centrality to lifestyle is related to a higher and sustained engagement in CS projects. The social dimension of the centrality concept, as well as the social dimension of the motivational construct, point in the same direction. Highly motivated and specialized birders may have their social lives centered on their leisure activity. Thus, birding itself is a lifestyle for these participants [31]. This may give a hint that the social dimension might be an important venue to recruit new people into existing CS projects.

However, enjoyment motivation and achievement motivation were unrelated to the different engagement clusters. This is an interesting aspect because it suggests that these motivational dimensions are not needed to engage people into CS programs with a high and sustained engagement. Furthermore, it shows that the enjoyment of nature is an important motivation, but this enjoyment is fulfilled even without a CS project. It further shows that this motivation does not decrease when people participate in more demanding CS projects. This is important because it suggests the even the CS programs with a high demand on time and skills do not decrease the outdoor motivation component.

The recreation perspective, especially the motivational dimensions of the Decker scale [11,29], probably do not take the full context of nature into account, and previous research suggests that in the context of biodiversity CS programs, concerns about nature and contribution to conservation are important motivations [18,19,20,21]. Therefore, future studies should link the recreation specialization concept with connectedness to nature, to learning, and to contributing to nature protection. The results confirm this, as the motivational dimensions enjoyment and achievement turn out to be unrelated to participation in CS programs. In addition, further motivational theories might be invoked to explain participation in CS programs, such as the self-determination theory, which suggests that feeling competent and experiencing autonomy are important drivers of human behavior [38]. However, social motivation was significantly different between the clusters, suggesting that social motivation is related to sustained engagement. This could be studied in combination with self-determination theory, where social relatedness is also an important dimension [38].

In conclusion, low-threshold projects for CS programs seem to attract participants when they have low participation obstacles, but keeping people within programs or moving them to a higher level of engagement might be easier when social dimensions (such as personal contact) are addressed because social motivation is higher in sustained engagement, with a high explained variance. Further studies should also accompany beginners with a panel design to predict retention within a program.

One last point should be made about the question of nest box activities. Here, it was not asked whether the respondents contributed to standardized nest box schemes or submitted the data for books, reports, or other scientific analyses; thus, further studies should explicitly ask the participants whether their nest box data were supplied to scientific research.

## Figures and Tables

**Table 1:** Participation in different CS programs. The first column shows the total number of participants. The following columns show the combination of programs combined by the birders.

	N Total	Winter Birds	Breeding Birds	Waterbirds	Ringing	Nest Boxes
Garden birds	1935 (68%)	1741	392	518	357	1050
Winter birds	1880 (66%)		380	519	353	1013
Breeding birds	597 (21%)			406	280	425
Waterbirds	800 (28%)				382	565
Ringing	558 (20%)					430
Nest boxes	1412 (49%)					

**Table 2:** Results of the multivariate model with the engagement cluster as the independent variable and birding specialization, involvement, and motivations as dependent variables.

Dependent Variable	Mean of Squares	F	*p*	Partial Eta-Squared
Involvement scale				
Attraction	87.391	144.301	**<0.001**	**0.098**
Centrality	291.149	299.167	**<0.001**	**0.183**
Social Bonding	187.811	307.532	**<0.001**	**0.187**
Identity	272.563	218.771	**<0.001**	**0.141**
Recreation Specialization				
Skill	308.172	588.860	**<0.001**	**0.306**
Behavior	175.909	477.155	**<0.001**	**0.263**
Personal commitment	126.391	157.986	**<0.001**	**0.106**
Behavioral commitment	106.237	253.600	**<0.001**	**0.160**
Motivations				
Social motivation	156.816	328.337	**<0.001**	**0.197**
Competition/Achievement	2.602	2.454	0.086	0.002
Enjoyment	12.120	36.678	**<0.001**	0.027

Note: In bold are *p* values < 0.001, and partial eta-squares above 0.05.

**Table 3:** Means (estimated from the linear model) of the three engagement clusters according to the different scales of specialization, involvement, and motivations. S = sustained engagement, L = low engagement, N = no engagement. *p* values between low and sustained engagement were all *p* < 0.001.

	No Engagement		Low Engagement		Sustained Engagement			
	Mean	SE	Mean	SE	Mean	SE	Post-Hoc Differences	*p* between Low and No
Involvement scale								
Attraction	3.65	0.03	3.84	0.02	4.30	0.03	S > L > N	*p* < 0.001
Centrality	2.49	0.03	2.71	0.03	3.64	0.04	S > L > N	*p* < 0.001
Social Bonding	2.05	0.03	2.20	0.02	2.96	0.03	S > L > N	*p* < 0.001
Identity	2.65	0.04	2.88	0.04	3.76	0.04	S > L > N	*p* < 0.001
Recreation Specialization								
Skill	−0.35	0.02	−0.25	0.02	0.78	0.03	S > L > N	*p* = 0.004
Behavior	−0.25	0.00	−0.21	0.02	0.59	0.02	S > L = N	*p* = 0.432
Personal commitment	2.35	0.03	2.55	0.03	3.12	0.03	S > L > N	*p* < 0.001
Behavioral commitment	1.49	0.02	1.60	0.02	2.17	0.02	S > L > N	*p* < 0.001
Motivations *								
Social	1.84	0.02	1.98	0.02	2.67	0.03	S > L > N	*p* < 0.001

* Note: Please note that competition motivation and enjoyment motivation were omitted because of non-significant results and a low effect size.

## Data Availability

On reasonable request from author.
